# Tooth Forms in Relation to Jaw Movements

**Published:** 1899-03

**Authors:** Alton H. Thompson

**Affiliations:** Topeka, Kan.


### Tooth Forms in Relation to Jaw Movements.

BY ALTON H. THOMPSON, D.D.S., TOPEKA, KAN.

Presented to the Section on Stomatology, at the Forty-ninth Annual Meeting ot the American Medical Association, held at Denver, Colo., June 7-10, 1898.

The writer feels like apologizing for addressing the Section upon such a simple thing as the relation of tooth forms to the movements of the jaws, and in defense makes the excuse that there is, as you well know, such a general misapprehension and lack of appreciation of many such simple principles of the profession that reiteration is never out of place. The writer has hopes to enlist the interest of the Section in the cultivation of a better appreciation of at least one of these simple principles. It is not sufficient to say that these things are found in the text-books. The text-books are rarely studied and the living principles must be presented over and over in meetings in various forms of dress, that all kinds of minds may be reached and interested and lifted up.

To be brief, therefore, (in a paper which can only be suggestive) we wish to call attention first to the causes of the various forms of jaws and teeth found in the animal kingdom. In following the teachings of evolution we must understand that function is the cause of structure, and that organs are created in response to demand for performance of functions. The teeth were therefore produced for the purpose of performing a function, and that function is the reduction and preparation of food for digestion. Not only were the teeth produced for this purpose primarily, but by the variations presented by different kinds of food animals came to employ, they have been infinitely modified and many various types evolved. This adaptive modification has led to the almost endless varieties of teeth and masticating apparatus found throughout the animal kingdom, all of which are adapted to the reduction of particular kinds of food. To the primary function of food reduction, there must be added the secondary offices that the teeth are often modified to perform, as tools, implements, weapons, etc.

The food-supply is one of the most potent influences in the environment of animals, and is secondary only to climatic conditions in determining the course and forms of life. The variation of the quantity or quality of the food-supply has led to the modification of all the species that have lived upon the earth. Gradual and persistent deviation has been the most potent for a change in structure, while sudden alterations in the food environment have resulted in the extinction of whole genera of mammals. The work of differentiation and conformatory adaptation of the masticating apparatus and teeth has progressed onward through the eons until the result is an extensive and most wonderful variation. But in all the variation and complexity of structure, we observe a remarkable adaptation of means to ends, of instruments to purpose, of tools to material. The cause is the material, the tool is the result. The variations in the food-supply caused the variations in tooth forms.

The same law applies also to the evolution of the jaws and masticating apparatus, the support and environment of the teeth, which were developed by adaptive modification for the same purpose, viz., the reduction of food preparatory to digestion. The teeth being merely the armament of the jaws, it follows that they were developed with them for the same purpose. The lower jaw or mandible has been described as a lever of the third class, in which the glenoid cavity is the fulcrum, the muscular force the power and the resistance of food the weight. Since the co-efficient of muscular power is 104 pounds per square inch, it is easy to surmise that the tendency of the exertion of the great masticating muscles upon the jaws and teeth must be to react powerfully upon those structures directly subjected to the resulting strain.

The principles of construction and motion of the specialized parts devoted to mastication and the precision and force with which this function is performed in the majority of animals possessing vertic mandibular occlusion, are wonderfully illustrative of the capacity of the animal mechanism for the display of power. The modification of the forms of the teeth and their supporting mechanism is directly due to the force of occlusion and of jaw movements. These tissues, like other parts, are disposed to develop in the line of greatest resistance in order to meet the demands of use. This is what Dr. John Ryder calls "displacement due to strain." He says, further, that "many of the parts associated in the function of mastication are greatly modified and brought to their present shape by the resistance incident to its performance. . . . There are distinct kinds of mandibular movement, and each kind corresponds to one very distinct type of tooth. Thus, in the carnivora, the tubercles are laterally compressed and raised into sharp edges to form effective cutting instruments, the teeth being developed in the direction of the vertic movement of the jaws." In the opposite extreme, the herbivora, the lateral movement of the mandible causes the plication of the molar crowns in a line transversely to the direction of the strain exerted during mastication. As the excursive movements have increased in complexity there has been increase in the complexity of the enamel foldings, ridges, crests, etc. It is concluded, therefore, that the various forms of teeth and mandibular articulations have been molded into shape by the jaw movements and force of occlusion, and also that they bear a direct relation to each other and exhibit associated types that are constant.

To go into some detail, we find that in the lowest and simplest form of tooth, that of the single cone, the reptilian type, it is associated with a mandibular movement that is purely vertic, mere opening and closing. This movement performs the one function of the teeth, i. e., prehension, for division and mastication of the food is unprovided for in these animals. There is wide opening of the jaws and after sharp closure a pulling motion is exercised. To resist these motions the teeth alternate above and below and are long, conic and sharp and slightly recurved to hold struggling prey. The teeth and jaws are well adapted to perform their functional duties together.

Among mammals, the two great extremes of types are the carnivora and herbivora, with some intermediate and composite types which subsist on an omnivorous diet. The carnivora, as you will recall, are possessed of a mandibular articulation which is like a simple hinge that allows of but one movement of the jaw, i. e., opening and closing, with no lateral excursion whatever. This movement requires the long canines and the long blades of the premolars to pass close to each other in the seizing and cutting of flesh, with a shear-like motion. The condyles are also shaped to resist the strong pulling motion which the curved canines are formed to effect in tearing flesh. The blades of the premolars and molars are developed vertically in the direction of greatest force, to effect the cutting of flesh. The jaw bones are short and stout and the closing muscles strongly developed to effect strong vertic force. The *Filtdæ* present this extreme modification, but there is a general departure from this type through the hyenas, dogs, bears and other omniverous animals which employ a mixed diet. The jaw articulation becomes more open, to allow of some lateral motion of the jaws; the teeth become modified to the tubercular form to masticate vegetable food, and the grinding teeth increase in number as the function of mastication comes more into use. In the Filidæ, the true cats, there are no tubercular teeth and no mastication, but in the more omniverous carnivora there is development of number and tubercularity of the teeth, and the jaws become longer.

In the other extreme, that of the herbivora or the planteaters, we have in the ungulata the best example of extreme lateral excursion of the mandible. This is due to the open temporo-maxillary articulation allowing extensive movements in various directions, but not much vertic play. There is also less density and diameter of the jaw but much lengthening. By a peculiar arrangement of the dental tissues, the wear of mastication preserves a constantly rough surface for the effective trituration of resisting vegetable fibers. The lateral movement of the jaws produces lateral extension of the teeth, in obedience to the law of development in the direction of resistance and use. The pigs show the first tendency to lateral movement and have lengthened crown crests to meet this movement in masticating. The tapir presents a little more lateral movement, and the kangaroo still more, with corresponding folding of the crown crests. The rhinoceros, the horse and their allies, have a larger jaw excursion, and the ruminants most of all. The forms of the molar crowns follow the law of displacement due to strain, and in the latter class present many very complex patterns, as in the ox, deer, sheep, etc., owing to foldings and complications of the crown crests. The movements of the jaw are lateral, anterior-posterior and diagonal, and in consequence the crowns of the molars are extended horizontally, with resisting ridges in every direction opposed to the horizontal movements. Other herbivora, as the rodenta, have complicated molar patterns also. Some, as the beaver, have molars composed of transverse plates of enamel, which are so arranged as to resist the anterior-posterior motion of the jaw, which is the main movement. This is also the main motion of the proboscidæ, the elephants, mastodon, etc., which have ridges of enamel arranged transversely to resist this main masticating movement.

In regard to man, who is a simple bunodont of primitive type, there are limited anterior-posterior and lateral jaw movements with slight crown crests and cusps to oppose them. Like the bears and most omnivorous animals, the crests are weak, and tubercles for mere crushing are the most marked feature of the occluding surfaces of the molars. The dentition of man is much like early types found in the eocene geologic formations, which were much generalized and from which the later more specialized forms were developed. Both his teeth and jaws are degraded if not primitive in type.

